# Supplementary material for: Definitions, Sources and Self-Reported Consumption of Regionally Grown Fruits and Vegetables in Two Regions of Australia
Source: Nutrients. 2020 Apr 8;12(4):1026. doi: 10.3390/nu12041026 (PMC7230907; doi:10.3390/nu12041026)
Supplement: Supplementary file 1 [file nutrients-12-01026-s001.pdf]

Table S1: Frequency of consumption of selected regionally grown fruit and vegetables TAS and SWA

		TAS				SWA				
	Portion	Monthly or less	Once a week	2-4 times a week	Daily or more	Monthly or less	Once a week	2-4 times a week	Daily or more	p-value
Asparagus, artichoke	1/2 cup	88 (76.5)	14 (12.2)	12 (10.4)	1 (0.9)	71 (77.2)	10 (10.9)	5 (5.4)	6 (6.5)	0.091
Asian greens	1/2 cup	82 (75.2)	16 (14.7)	10 (9.2)	1 (0.9)	-	-	-	-	-
Bean, peas	1/2 cup	21 (18.1)	25 (21.6)	50 (43.1)	20 (17.2)	50 (53.8)	16 (17.2)	21 (22.6)	6 (6.5)	<0.001
Beetroot	1/2 cup	75 (65.2)	21 (18.3)	16 (13.9)	3 (2.6)	-	-	-	-	-
Broccoli, brussels sprouts, cauliflower, cabbage	1/2 cup	9 (7.7)	18 (15.4)	58 (49.6)	32 (27.4)	11 (11.7)	13 (13.8)	43 (45.7)	27 (28.7)	0.761
Carrots, parsnips, turnips, swede, fennel	1/2 cup	5 (4.3)	17 (14.7)	48 (41.4)	46 (39.7)	12 (12.8)	16 (17.0)	47 (50.0)	19 (20.2)	0.008
Capsicum	1/2 cup	23 (19.7)	41 (35.0)	40 (34.2)	13 (11.1)	28 (30.8)	29 (31.9)	26 (28.6)	8 (8.8)	0.319
Celery	1/2 cup	50 (43.5)	32 (27.8)	27 (23.5)	6 (5.2)	50 (54.3)	22 (23.9)	15 (16.3)	5 (5.4)	0.415
Corn	1/2 cup	43 (38.1)	37 (32.7)	29 (25.7)	4 (3.5)	58 (61.7)	17 (18.1)	12 (12.8)	7 (7.4)	0.001
Cucumber	1/2 cup	34 (30.1)	28 (24.8)	38 (33.6)	13 (11.5)	-	-	-	-	-

Leeks, onions, shallots, spring onion	1/2 cup	11 (9.8)	16 (14.3)	52 (46.4)	33 (29.5)	27 (29.7)	15 (16.5)	34 (37.4)	15 (16.5)	0.002
Leafy greens	1 cup fresh or ½ cup cooked	9 (7.8)	8 (7.0)	59 (51.3)	39 (33.9)	16 (17.4)	18 (19.6)	32 (34.8)	26 (28.3)	0.003
Mushrooms	1/2 cup	33 (29.2)	35 (31.0)	39 (34.5)	6 (5.3)	-	-	-	-	-
Potatoes	1/2 cup	13 (11.5)	18 (15.9)	64 (56.6)	18 (15.9)	17 (18.3)	25 (26.9)	33 (35.5)	18 (19.4)	0.021
Pumpkin	1/2 cup	37 (32.5)	37 (32.5)	34 (29.8)	6 (5.3)	31 (33.3)	24 (25.8)	28 (30.1)	10 (10.8)	0.427
Radishes	1/2 cup	98 (89.9)	5 (4.6)	5 (4.6)	1 (0.9)	-	-	-	-	-
Tomatoes	1/2 cup	12 (10.7)	22 (19.6)	56 (50.0)	22 (19.6)	13 (14.0)	20 (21.5)	38 (40.9)	22 (23.7)	0.607
Zucchini, squash, eggplant	1/2 cup	38 (34.5)	24 (21.8)	38 (34.5)	10 (9.1)	39 (31.7)	26 21.1	20 (16.3)	7 (5.7)	0.125
Herbs and spices	1 tablespoon	12 (10.6)	13 (11.5)	32 (28.3)	56 (49.6)	23 (25.6)	17 (18.9)	31 (34.4)	19 (21.1)	<0.001
Apples, pears	1 medium	12 (10.7)	12 (10.7)	35 (31.3)	53 (47.3)	17 (19.1)	13 (14.6)	31 (34.8)	28 (31.5)	0.097
Stone fruits	1 medium or 2 small	48 (42.5)	25 (22.1)	24 (21.2)	16 (14.2)	41 (47.1)	16 (18.4)	22 (25.3)	8 (9.2)	0.587

Lemons	¼ lemon or 1 tb juice	43 (38.4)	33 (29.5)	24 (21.4)	12 (10.7)	29 (33.3)	16 (18.4)	23 (26.4)	19 (21.8)	0.066
Berries, cherries	1/2 cup	31 (27.4)	32 (28.3)	28 (24.8)	22 (19.5)	27 (30.7)	18 (20.5)	26 (29.5)	17 (19.3)	0.608
Passionfruit	1 medium or 2 small	95 (88.8)	8 (7.5)	2 (1.9)	2 (1.9)	-	-	-	-	-
Figs	1 medium or 2 small	96 (88.9)	9 (8.3)	1 (0.9)	2 (1.9)	-	-	-	-	-
Quince, rhubarb	½ cup cooked	98 (88.3)	8 (7.2)	4 (3.6)	1 (0.9)	-	-	-	-	-
Avocado	½ medium	-	-	-	-	28 (32.2)	18 (20.7)	23 (26.4)	18 (20.7)	-
Citrus	1 medium	-	-	-	-	20 (22.5)	19 (21.3)	31 (34.8)	19 (21.3)	-
Grapes	1 cup	-	-	-	-	56 (64.4)	13 (14.9)	14 (16.1)	4 (4.6)	-
Kiwi fruit	2 small	-	-	-	-	65 (74.7)	11 (12.6)	8 (9.2)	3 (3.4)	-
Persimmons	1 medium	-	-	-	-	82 (94.3)	2 (2.3)	2 (2.3)	1 (1.1)	-
Prunes	½ cup	-	-	-	-	79 (89.8)	4 (4.5)	4 (4.5)	1 (1.1)	-

Berries (including blackberry, blueberry, gooseberry, raspberry, strawberry, tayberry, yosterberry)

Stone fruits (including apricot, greengage, nectarine, peach, plum)

Herbs (including chilli, garlic, thyme, parsley, coriander, rosemary, oregano, chives, basil, sage, mint

Leafy greens (including lettuce, rocket, sprouts chard/silver beet, kale, spinach, mustard greens)

p-value derived from chi-square test
